# DeepRegFinder: deep learning-based regulatory elements finder

**DOI:** 10.1093/bioadv/vbae007

**Published:** 2024-01-14

**Authors:** Aarthi Ramakrishnan, George Wangensteen, Sarah Kim, Eric J Nestler, Li Shen

**Affiliations:** Friedman Brain Institute and Nash Family Department of Neuroscience, Icahn School of Medicine at Mount Sinai, New York, NY 10029, United States; Department of Computer Science, Brown University, Providence, RI 02912, United States; Cancer Program, Broad Institute, Cambridge, MA 02142, United States; Friedman Brain Institute and Nash Family Department of Neuroscience, Icahn School of Medicine at Mount Sinai, New York, NY 10029, United States; Friedman Brain Institute and Nash Family Department of Neuroscience, Icahn School of Medicine at Mount Sinai, New York, NY 10029, United States

## Abstract

**Summary:**

Enhancers and promoters are important classes of DNA regulatory elements (DREs) that govern gene expression. Identifying them at a genomic scale is a critical task in bioinformatics. The DREs often exhibit unique histone mark binding patterns, which can be captured by high-throughput ChIP-seq experiments. To account for the variations and noises among the binding sites, machine learning models are trained on known enhancer/promoter sites using histone mark ChIP-seq data and predict enhancers/promoters at other genomic regions. To this end, we have developed a highly customizable program named DeepRegFinder, which automates the entire process of data processing, model training, and prediction. We have employed convolutional and recurrent neural networks for model training and prediction. DeepRegFinder further categorizes enhancers and promoters into active and poised states, making it a unique and valuable feature for researchers. Our method demonstrates improved precision and recall in comparison to existing algorithms for enhancer prediction across multiple cell types. Moreover, our pipeline is modular and eliminates the tedious steps involved in preprocessing, making it easier for users to apply on their data quickly.

**Availability and implementation:**

https://github.com/shenlab-sinai/DeepRegFinder

## 1. Introduction

DNA regulatory elements (DREs) are genomic regions that play a crucial role in controlling gene expression by interacting with chromatin and DNA binding proteins. DREs can be broadly classified into four major categories, namely promoters, enhancers, silencers, and insulators (Riethoven 2010, Chatterjee and Ahituv 2017, Doane and Elemento 2017), with promoters and enhancers being the most extensively researched. Promoters are DREs located proximal to the transcriptional start sites (TSSs) of genes, typically spanning 0.1 to 1 kb. They facilitate transcription initiation by interacting with RNA polymerase and other transcription factors (TFs) (Haberle and Stark 2018, Le *et al.* 2019). Enhancers on the other hand are DREs that can act over long distances to stimulate gene expression (Li *et al.* 2019, Panigrahi and O’Malley 2021). They are typically up to 1 Mb away from the TSSs of the genes being regulated (Pennacchio *et al.* 2013). Enhancers are cell-type-specific and are involved in development and disease (Spitz and Furlong 2012, Parker *et al.* 2013). They coordinate with promoters via formation of DNA loops to mediate gene expression (Arnold *et al.* 2020).

Identification of enhancers is a critical task as it has multiple implications: enhancers are known to regulate cell-type-specific gene expression in the body (Andersson *et al.* 2014); it is important for identifying therapeutic targets as changes in enhancer activity can give rise to diseases (Chatterjee and Ahituv 2017); it helps researchers gain a deeper understanding of the genetic mechanisms underlying disease and develop more targeted and effective treatments (Pennacchio *et al.* 2013); it helps to shed light on the function of non-coding regions of the genome, which still remain poorly understood (Perenthaler *et al.* 2019).

Both enhancers and promoters display distinctive chromatin modification patterns (Calo and Wysocka 2013) which can be derived from ChIP-seq data. However, due to the variations among the DREs and the intrinsic noises of high-throughput experiments, it remains a challenge to accurately identify them. It is relatively easy to tell apart DREs from other genomic regions, i.e. the background, based on the enrichment of certain histone marks. But it is more difficult to classify different types of DREs, such as enhancers and promoters. Several machine learning methods have been developed to train on known enhancers and promoters to predict the unknown DREs across the genome. Segmentation and genome annotation algorithms (Ernst and Kellis 2017, Girimurugan *et al.* 2018, Libbrecht *et al.* 2021) employ probabilistic models to segment the genome and annotate them as promoters, enhancers or genes. But the existing methods are often difficult to use, especially in preprocessing the raw data to be ready for consumption by the programs. They also tend to single out enhancers as the positive class and group everything else, including promoters, into the negative class, which makes it difficult to assess the performance in a real-world setting.

In this study, we present DeepRegFinder—a customizable computational pipeline that allows one to process ChIP-seq data efficiently and apply deep learning models to predict DREs using a user-friendly command line interface. DeepRegFinder presents two models to choose from for DRE prediction—Convolutional Neural Network (CNN) and Recurrent Neural Network (RNN) (LeCun *et al.* 2015). A CNN uses convolutional filters as feature extractors to capture ubiquitous patterns in the input and a stack of convolutional layers to build up a feature hierarchy. The features at the highest level of the hierarchy are subsequently used to make predictions on the input. An RNN extracts features from each position of an input sequence, combines them with features from the previous position to produce new features for the current position. An RNN can use these features to make predictions at each position or at the end of the input sequence. Each model has its own merits and limitations: CNNs excel at learning spatial-invariant feature hierarchies, e.g. the chromatin modification motifs and their combinations indicative of DREs that may occur at different locations of an input. But CNNs may not be able to detect long-range interactions of the motifs. In contrast, some variants of RNNs, such as the long-short term memory (LSTMs) networks (Hochreiter and Schmidhuber 1997), have built-in mechanisms to learn long-range interactions by retaining in memory the information learned from previous positions in the sequence to make future predictions. Multiple LSTMs can also be stacked to learn complicated features from inputs. For this reason, RNNs are very effective models for sequential data (LeCun *et al.* 2015) such as DNA sequences. But they are known to be more difficult than CNNs to optimize. In DeepRegFinder, we provide an option for the user to decide between the two models.

DeepRegFinder offers users the option to run three different types of classifications. Firstly, two-class classification distinguishes enhancers from background genomic regions, including both generic background and promoter regions. Secondly, three-class classification classifies enhancers, promoters, and generic background regions. Thirdly, five-class classification can further classify enhancers and promoters into active and poised states for a given cell type (Creyghton *et al.* 2010, Rada-Iglesias *et al.* 2011), a feature which most existing tools lack. The five-class classification therefore classifies any genomic region into active and poised enhancers (AEs and PEs, respectively), active and poised promoters (ATs and PTs, respectively), and background (Bgd). The enhancer class in two-class classification includes both active and poised enhancers. Similarly, enhancer and promoter classes in three-class classification include both active and poised states. The active or poised states are defined using read coverage derived from GRO-seq (Core *et al.* 2008), PRO-seq (Kwak *et al.* 2013), NET-seq (Churchman and Weissman 2011), or any kind of sequencing techniques that measure the transcriptional activity of a DRE. It is important to accurately identify enhancers/promoters belonging to active and poised states as it provides information on the DREs that are indeed functional in a given cell type or condition. We compare our methods with existing algorithms in both three-class and five-class classification tasks and find DeepRegFinder to always achieve better performance.

## 2. Methods

DeepRegFinder trains deep neural networks in a supervised fashion to identify enhancers and promoters. It comprises three modules: Preprocessing, Training, and Prediction. Each module can be run independently, providing flexibility for users to train a model once and use it for prediction multiple times. A brief description for each module is provided below.

### 2.1 Preprocessing module

The preprocessing module obtains read coverage for histone marks and TFs at promoters, enhancers, and background genomic regions to generate training, validation, and test datasets. Users may download processed alignment files (BAM) for ChIP-seq data from the ENCODE (ENCODE Project Consortium 2012) website for a variety of histone marks such as H3K27ac, H3K4me1, H3K4me3, or use their own alignment files. Promoters are defined based on user provided TSS annotation files (BED), wherein each site is slopped to 2 kb. TSS annotation files can be easily obtained from websites such as Ensembl https://www.ensembl.org/, UCSC Genome Browser https://genome.ucsc.edu/, or GENCODE https://www.gencodegenes.org/.

To determine promoters that are accessible for a given cell line, DNase I hypersensitive site (DHS) or Assay for Transposase-Accessible Chromatin with sequencing (ATAC-seq) data obtained from the same cell line are utilized to intersect with the TSS annotations. Enhancers are defined using user-provided peak lists for enhancer-specific TFs such as p300 and CBP. The TF peaks are used to intersect with DHS or ATAC-seq peaks and subtract H3K4me3 sites or TSSs to avoid any overlap with promoters. These regions are slopped to 2 kb as well. For defining background, a total of 30 000 genomic regions of 2 kb length are selected randomly, after the exclusion of enhancers, promoters, DHS sites, and TF peaks. A detailed explanation of the definition of promoters, enhancers, and background genomic regions can be found in the Preprocessing Module section of the Supplementary Information File.

To define the input features, the entire genome is divided into windows of 2 kb in size. Each window is further divided into 20 bins of 100 bp each. The window size, number of bins, and bin size are all configurable parameters of the preprocessing module. Read counts of histone marks from user-provided BAM files for enhancers, promoters and background regions are obtained for all 100 bp bins of the genome using featureCounts (Liao *et al.* 2014). The read counts are normalized to Reads Per Million mapped reads values, which are further averaged across all replicates for each histone mark (see “Genomic binning and ChIP-seq processing” section of Supplementary Information). In the case of five-class classification, enhancers and promoters are further divided into poised and active states using GRO-seq (or similar technique) data. GRO-seq experiments map binding sites of RNA polymerase II that are transcriptionally active. Users may provide GRO-seq data to DeepRegFinder in the form of BAM files. The coverage of GRO-seq is obtained for all enhancer and promoter sites by the preprocessing pipeline, and K-means clustering is applied on the GRO-seq coverage to classify them into active and poised states. The coverage of the final enhancers, promoters and random background regions are combined into a 3D tensor of sites × histone marks × bins consisting of the normalized read counts for each region. In the case of 3 class classification, background, enhancer, and promoter regions are assigned class labels 0, 1, and 2 respectively, whereas in the case of five-class classification, the Bgd, PE, AE, PT, and AT are assigned class labels 0, 1, 2, 3, and 4, respectively. This tensor is ultimately divided into training, validation, and test sets to be used in the training module. The split is based on chromosome names (by default) or randomly. Details on the train-validation-test split can be found in the “Creation of training, validation and test set” sub-section of the Preprocessing Module section in the Supplementary Information File. For this study, we obtained data for dozens of histone marks across three different cell lines: K562, GM12878, and HepG2 from the ENCODE website. A complete list of the histone marks used for each cell line is provided in Supplementary Table S4. We also obtained TF peak lists from the ENCODE website and they are listed in Supplementary Table S5.

### 2.2 Training module

The training module trains either a CNN or RNN model on the training set, uses the validation set to save the best model and evaluates on the testing set. Users can specify training-related parameters such as learning rate, number of epochs and batch size.

#### 2.2.1 Neural network architectures

**Figure 1.**
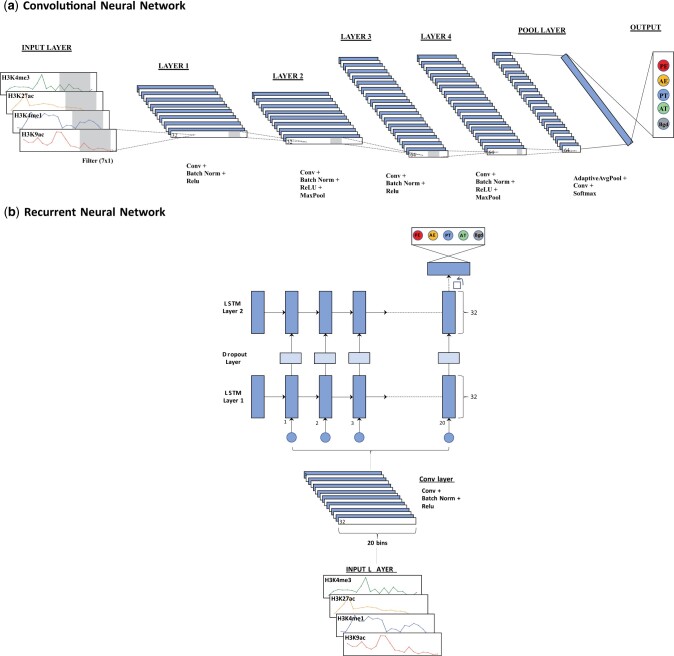
Network structures for (a) CNN. The input for CNN is a tensor consisting of the normalized read counts for each histone mark across bins. The first four convolutional layers are followed by a batch normalization layer as well as a ReLU activation layer. Max pooling is applied for layer 2 and 4. The final pooling layer consists of a global average pooling followed by a 1D convolutional layer and a softmax layer for classification. (b) RNN. Input for the RNN is the same as that for the CNN. It includes a 7 × 1 1D convolutional layer at the bottom which is followed by two LSTMs with the size of 32. They are followed by a softmax layer for classification.

The structures of the CNN and RNN are presented in Fig. 1. We tested several architectures for the CNN through multiple rounds of trial and error and arrived at a model with five convolutional layers. The CNN model incorporates a 7 × 1 1D convolutional layer at the bottom, serving as a feature extractor to detect histone modification patterns. This layer functions as a “motif” detector that extracts binding patterns from the read coverage (represented in gray in Fig. 1). Each of the first four convolutional layers is followed by a batch normalization (Ioffe and Szegedy 2015) layer as well as a ReLU (Nair and Hinton 2010, Dahl *et al.* 2012) activation layer. The batch normalization layer speeds up learning and prevents overfitting by reducing internal covariate shift. Padding is used to preserve spatial resolution through successive convolutional layers. Max pooling is applied for Layers 2 and 4 to reduce the spatial resolution of the feature maps. The final pooling layer consists of a global average pooling followed by a 1D convolutional layer and a softmax layer for classification. The RNN model was also determined by trial and error. It includes a 7 × 1 1D convolutional layer at the bottom, followed by two stacked LSTMs with the size of 32. They are followed by a softmax layer for classification. To address overfitting, a dropout layer is incorporated in the RNN model.

#### 2.2.2 Network training

Both CNN and RNN use Adam (Kingma and Ba 2014) as the optimizer. We chose a learning rate of 0.01 for our models because a higher learning rate would result in faster convergence but increase the risk of divergence, while a lower learning rate would require more training epochs to reach convergence. The weight decay was set to 0.0001 as this value yielded the best performance. We employed the negative log likelihood loss (i.e. multinomial cross-entropy loss) for both models. The *ReduceLROnPlateau* learning rate scheduler was utilized with *mode* set to “max” for maximizing the mean average precision (mAP) for each epoch and the parameters *factor* and *patience* set to 0.1 and 5, respectively. The CNN and RNN in DeepRegFinder are both parameter efficient with only 26K and 12K weights and biases, respectively. Both models were implemented using PyTorch (Paszke *et al.* 2019).

For different cell lines, the sizes of active/poised enhancer/promoter classes vary a lot (Supplementary Table S3). Additionally, we chose to make the background class larger than the non-background classes to represent the diverse genomic background regions. In order to address the issue of class imbalance, we implemented a weighted sampler to construct balanced training batches. Throughout the training process, the model is evaluated on the validation set every 1000 batches. If the performance of the model is greater than that from a previous evaluation, the current model is saved. We found that training for 10 epochs was sufficient to achieve the best performance. The saved best model will be used for making predictions on the test set. The total training time typically ranged from 5 to 10 min, depending on the user-specified number of epochs and bin size. After training, the training module generates a report that includes precision–recall values for each class along with ROC curves, PR curves, and confusion matrices based on the test set.

### 2.3 Prediction module

The prediction module utilizes the best model to classify each of the 2 kb windows that cover the entire genome in a step size of 200 bps. The entire genome’s read coverage data are obtained during the preprocessing stage. The predictions that pass a probability cutoff of 0.5 for a non-background class are stored as the filtered predictions. All the filtered predictions are then consolidated by grouping adjacent predictions of the same class into larger blocks. The validation rates for consolidated enhancers and promoters are calculated to obtain an estimate of the model’s false positive rate by overlapping them with positive markers (PMs). Information regarding the definition of PMs is provided in the “Defining Positive Markers” subsection of the Preprocessing Module section in the Supplementary Information File.

### 2.4 Creation of training, validation, test set and external validation set

At the end of the preprocessing pipeline, the samples consisting of normalized read coverage of enhancers, promoters and randomly selected background genomic regions are merged into a single set. Each sample in this set is a 2 kb region and represents the normalized read coverage for all histone marks. A 3D tensor of dimension samples × histone marks × bins consisting of the normalized read counts for each region is constructed. This 3D tensor is divided into training, validation and test sets. The user can choose between two different modes for creating the train-validation-test split. The first mode uses a chromosome-wise split of the training, validation, and test sets, i.e. samples belonging to a chromosome will only be assigned to one of the datasets. The second mode randomly splits the samples into training, validation, and test set in the percentage ratio of 60:20:20. For this study, we use the chromosome-wise split. We include regions from chromosome 1–7 in the training set, chromosome 8–16 in the validation set and chromosome 17–22, X and Y in the test set. The total numbers of samples for each class across the three cell lines are listed in Supplementary Table S3.

As a baseline for machine learning based methods, we downloaded enhancer and promoter annotations from two public databases—SCREEN https://screen.encodeproject.org/ and EnhancerAtlas 2.0 http://www.enhanceratlas.org/—to compare against the test set. Both databases use knowledge-based, *ad hoc* rules to identify potential DREs on the genome. SCREEN candidate cis-Regulatory Elements (cCREs) were obtained for K562, GM12878, and HepG2 cell line. Only the sites labeled as distal enhancer-like signatures and promoter-like signatures were retained for the comparison. Obtaining BED file of enhancers from EnhancerAtlas database was a straightforward process. We converted the coordinates of EnhancerAtlas database from hg19 to hg38 using the CrossMap Python package (Zhao *et al.* 2014). The number of sites used for both databases are included in Supplementary Table S13. For comparison with the test set, we used the command “bedtools intersect” to find an overlap between the test set sites of DeepRegFinder and the SCREEN and EnhancerAtlas sites

For further validation of DeepRegFinder’s predictions, we obtained 664 experimentally validated enhancer-gene pairs as well as the negative control regions reported in (Gasperini *et al.* 2019). We converted the hg19 coordinates to hg38 using the CrossMap Python package (Zhao *et al.* 2014).

## 3. Results

### 3.1 Model evaluation using three-class classification

We conducted a comparative analysis of DeepRegFinder against five established methods, namely Random-Forest Based Algorithm for Enhancer Identification from Chromatin State (RFECS) (Rajagopal *et al.* 2013), enhancer HMM (eHMM) (Zehnder *et al.* 2019), Probabilistic Enhancer PredictIoN Tool (PREPRINT) (Osmala and Lähdesmäki 2020), Enhancer Prediction using Deep Neural Network (EP-DNN or KimNet) (Kim *et al.* 2016), and ChromHMM (Ernst and Kellis 2017) (see Table 1 for description of each tool). The data preprocessing for each method is provided in the “Comparison of DeepRegFinder with existing tools” section of the Supplementary Information File. Because these methods cannot distinguish active and poised enhancers/promoters, we employed the three-class classification mode of DeepRegFinder. For ChromHMM, we manually assigned the chromatin states learned on the three cell lines (Supplementary Fig. S3) to the three classes since it is a purely unsupervised method.

**Table 1.** Summary of all tools utilized in this study.

Name	Reference	Methodology
ChromHMM	Ernst and Kellis (2017)	Unsupervised HMM + manual labeling
RFECS	Rajagopal *et al.* (2013)	Random Forest
EP-DNN	Kim *et al.* (2016)	Multi-layer perceptron
PREPRINT	Osmala and Lähdesmäki (2020)	Bayesian derived probabilistic scores + SVM with Gaussian kernel
eHMM	Zehnder *et al.* (2019)	Supervised HMM

**Figure 2.**
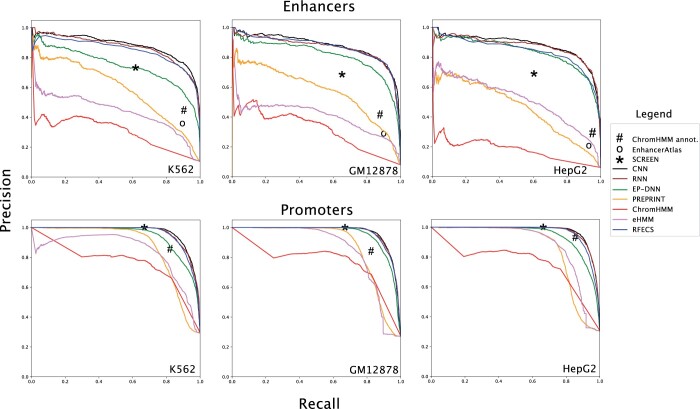
Precision–recall curves for three-class classification. Precision–recall curves to compare the performance of 7 different methods for enhancers and promoters for K562, GM12878, and HepG2 cell lines.

Overall, both CNN and RNN models of DeepRegFinder compare favorably with the other methods in precision and recall scores on the test set across all cell types (Fig. 2 and Supplementary Table S1). Only RFECS achieves performance close to the two models, followed by EP-DNN (i.e. KimNet). The other three methods are significantly worse, with ChromHMM consistently ranked at the bottom. For enhancer prediction, the precision is in the range of 0.71–0.81, while the recall is in the range of 0.84–0.93 for the two DeepRegFinder models. For promoter prediction, the precision is in the range of 0.91–0.95, while the recall is in the range of 0.86–0.89 for the two DeepRegFinder models. Further analysis through the confusion matrices of the two models (Supplementary Fig. S1) reveals a small amount of misclassifications between the promoter and enhancer classes. Only a tiny fraction of the enhancers and promoters are classified as background and vice versa. The performance of the RNN model is on par with that of the CNN model; both reach mean average precision (mAP) in the range of 0.91–0.93 (Supplementary Table S1).

Performance of the other methods are highly variable, especially on enhancer prediction. For instance, EP-DNN demonstrates poor performance for enhancer identification on the K562 cell line in comparison to DeepRegFinder. However, its performance is more in line with that of DeepRegFinder on the GM12878 and HepG2 cell lines. On the other hand, the method consistently demonstrates good performance for identifying promoters across all cell lines, with slightly lower precision and recall than that of DeepRegFinder. Both PREPRINT and eHMM’s performance in enhancer prediction are significantly lower than that of DeepRegFinder, RFECS, and EP-DNN. PREPRINT is notably better than eHMM on the K562 and GM12878 cell lines but slightly worse on the HepG2 cell line. ChromHMM is significantly worse than all the other methods in both enhancer and promoter prediction across all cell lines. We hypothesize that ChromHMM as a fully unsupervised method requires much more training data than its supervised counterparts for DRE classification. We did an additional analysis to compare the publicly available ChromHMM annotations based on a model trained with 127 cell lines https://egg2.wustl.edu/roadmap/web\_portal/chr\_state\_learning.html with our test set. We found this version of ChromHMM to perform better than PREPRINT, eHMM and the ChromHMM but still worse than DeepRegFinder, especially for enhancer prediction (Fig. 2 and Supplementary Table S6). All methods achieve better performance in promoter prediction than enhancer prediction, showing that the enhancers are more difficult to identify than promoters in general.

To provide additional baselines for model performance, we used the enhancers and promoters defined in SCREEN and EnhancerAtlas databases. Both databases represent collections of candidate enhancers and promoters based on *ad hoc* rules. We found SCREEN’s promoter annotation to be in line with that of DeepRegFinder but enhancer annotation to be below the PR curve of DeepRegFinder but still above that of PREPRINT, eHMM and ChromHMM (Fig. 2 and Supplementary Table S10). EnhancerAtlas’s enhancer annotation has a recall of around 0.9 but a precision of only 0.20–0.35 across the three cell lines (Fig. 2 and Supplementary Table S11). This analysis provides some baseline performance of an enhancer and promoter database that is based on biological knowledge and simple statistical analysis rather than complicated machine learning models.

We further compared the predicted enhancers from all tools with the 664 experimentally validated enhancers and negative control regions from (Gasperini *et al.* 2019) (Supplementary Table S12). DeepRegFinder (CNN), eHMM and RFECS show high precision of >0.95 but both eHMM and RFECS have low recall of around 0.6, while DeepRegFinder (CNN) has a recall of 0.85. EP-DNN, DeepRegFinder (RNN), and PREPRINT show high recall at or above 0.95 but relatively lower precision. Judging by the F1 scores, DeepRegFinder (CNN and RNN), EP-DNN, and PREPRINT achieve the top spots with DeepRegFinder (RNN) ranks at the top, followed by PREPRINT and DeepRegFinder (CNN). ChromHMM has high precision of 0.92 but low recall of 0.40, which make it again rank at the bottom among all tools in terms of F1 score. It shall be noted that the experimentally validated enhancers (Gasperini *et al.* 2019) are only a small subset of all enhancers on the genome and can be biased in terms of their chromatin binding characteristics due to the small sample size. We also inspected the validated enhancers that were not recalled as enhancers for each tool and found the majority of them to be predicted as promoters (Supplementary Table S12). This is in line with the confusion matrix analysis (Supplementary Fig. S1) that enhancers are more likely to be predicted as promoters than background.

Lastly, we computed the validation rates of enhancers and promoters predicted by DeepRegFinder’s prediction module by overlapping them with PMs (Supplementary Table S8). The validation rates are all above 0.95 for both promoters and enhancers across all cell lines.

### 3.2 Model evaluation using five-class classification

In addition to three-class classification, DeepRegFinder can perform five-class classification. Using this mode, our program can distinguish between poised and active states of enhancers and promoters. Poised enhancers are often identified by the decreased level of H3K27ac and the enrichment of H3K27me3 histone marks (Rada-Iglesias *et al.* 2011). On the contrary, active enhancers are often characterized by the increased level of H3K27ac and the absence of H3K27me3 (Gray *et al.* 2015). Both poised and active enhancers contain the presence of H3K4me1. While active enhancers are those regions that are actively involved in the regulation of gene expression under normal conditions, poised enhancers can transition to active states in response to specific pathways and developmental cues (Creyghton *et al.* 2010). It is therefore important to accurately identify both active and poised states of enhancers. Similarly, poised and active promoters contain the presence of H3K27me3 and H3K9ac, respectively. While both contain the enrichment of H3K4me3. The distinction between the poised and active states of promoters and enhancers in histone modifications are not as strong as the distinction between promoter and enhancer, or between enhancer and background, which makes it a more challenging task. Although no other method is designed to distinguish active and poised states of enhancers/promoters, we implemented our own versions of EP-DNN and RFECS to make them perform five-class classifications.

**Figure 3.**
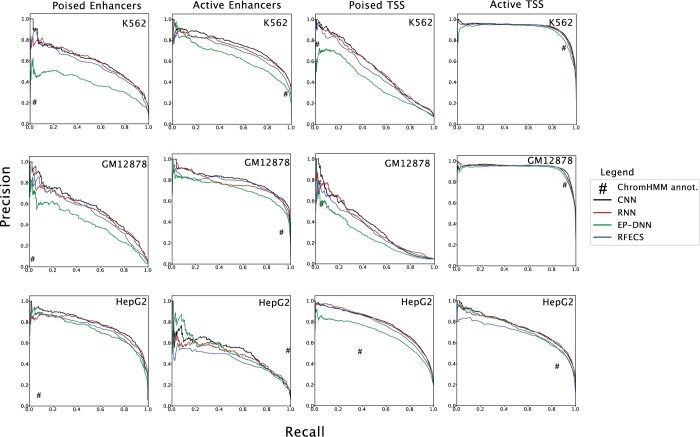
Precision–recall curves for five-class classification. Precision–recall curves to compare the performance of DeepRegFinder, RFECS and EP-DNN for Poised Enhancers (PE), Active Enhancers (AE), Poised TSS (PT), and Active TSS (AT) for K562, GM12878, and HepG2 cell lines.

Comparing the precision and recall of the four models on the test set (Fig. 3 and Supplementary Table S2), both the CNN and RNN models of DeepRegFinder consistently outperform EP-DNN and RFECS across all classes and all cell types. The mAP scores of CNN and RNN varies between 0.65 and 0.71 for the three cell types. Again, the performance of RNN is on par with that of CNN. There is no clear winner between EP-DNN and RFECS: while RFECS defeats EP-DNN on the K562 and GM12878 cell lines, it slightly underperforms EP-DNN on the HepG2 cell line. We also performed the analysis of comparing the publicly available ChromHMM annotations with the five-class labels of DeepRegFinder and found its performance to vary wildly across classes and cell lines (Fig. 3 and Supplementary Table S7). Although it is sometimes in line with the other models, especially on the active promoter, its scores can be well below the curves of the other models. The only exception is on the active enhancer in the HepG2 cell line where it outperforms all the other models, including DeepRegFinder. We note that the active enhancer class in the HepG2 cell line is unusually small in comparison with other cell lines (Supplementary Table S3). This has caused performance degradation for all models but does not affect ChromHMM annotations.

Comparing the four non-background classes, all models achieve the best performance on active promoter on the K562 and GM12878 cell lines, with precision and recall exceed or close to 0.9 for both CNN and RNN. The performance of active promoter is on par with poised enhancer and promoter on the HepG2 cell line. The ranking of the performance on the other three non-background classes varies depending on the cell types. This shows that the active promoter class has the most distinctive histone modification profiles among the four classes to make them easiest to identify. Confusion matrix analysis (Supplementary Fig. S2) shows that poised enhancer is most likely to be misclassified as active enhancer, followed by poised promoter. Active enhancer is most likely to be misclassified as poised enhancer, followed by active promoter. Poised promoter is most likely to be misclassified as active promoter or background. On the HepG2 cell line, there is an elevated likelihood for the active promoter to be misclassified as poised promoter.

**Figure 4.**
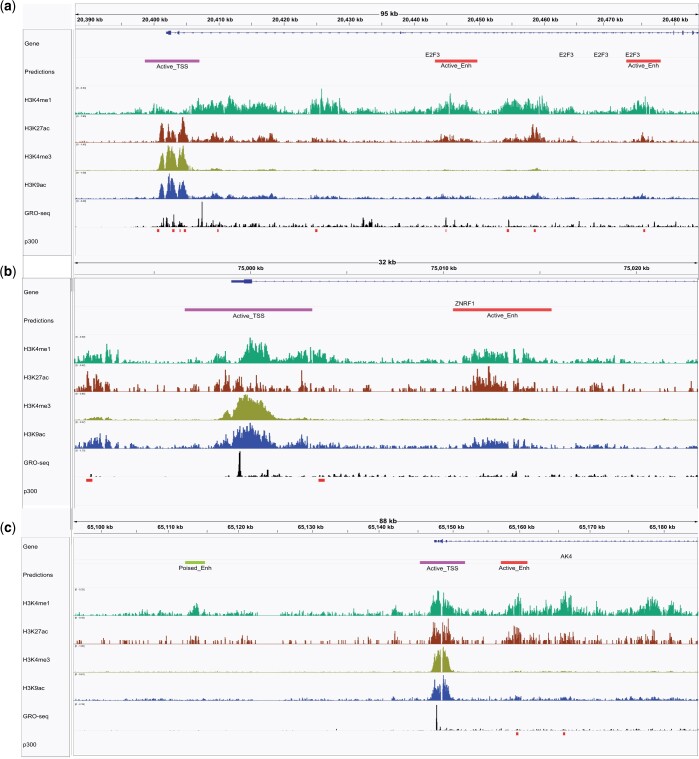
Genome browser screenshots. Example screenshots of genome browser displaying DeepRegFinder CNN’s predictions in the K562 cell line.

Figure 4 represents three genome browser screenshots of DeepRegFinder’s predictions in the K562 cell line using the CNN model. It demonstrates that the predicted active promoters contain the characteristic enrichment of H3K27ac, H3K4me3, and H3K9ac; the predicted active enhancers contain the enrichment of H3K4me1, H3K27ac and the depletion of H3K4me3; the predicted poised enhancer located upstream of the AK4 gene contains the enrichment of H3K4me1 but is depleted with the activating histone marks.

Lastly, we computed the validation rates of poised/active enhancers and promoters predicted by DeepRegFinder’s prediction module (Supplementary Table S9). The validation rates are mostly above 0.95 for all classes across all cell lines. Only poised enhancer and poised promoter sometimes show slightly lower validation rate in the range of 0.93–0.95.

### 3.3 Activation heatmap and weight matrices of the first convolution layer filters

One of the advantages of using a convolutional layer as the first layer of the CNN and RNN is that the convolutional filters of the first layer (Fig. 1a—Layer 1) can be interpreted as feature detectors for “chromatin motifs”. The combinations of such motifs are then learned by the following layers of the model for classification. To demonstrate the chromatin motifs learned by the first layer, we selected the top 100 regions for each of the five classes in the test set by their predicted class probabilities, resulting in a total of 500 regions. Each region is represented by an array with dimension of 20 × 12 that represents the coverage across 20 bins for 12 histone marks. In convolution, this region is known to have a length of 20 and a depth of 12, where each dimension of the depth is also known as a channel. The selected regions were passed through a CNN trained on the K562 cell line to obtain the activations of the first convolutional layer. The first convolutional layer contains 32 filters, each of dimension 7 × 12 that represents the weights spanning 7 bins and across 12 channels (i.e. histone marks). A filter scans the input to derive activation values by computing the dot product between the filter and a segment of 7 bins of the input region. The convolution is designed in a way so that the output has the same length as the input by padding appropriate zero values on both ends of the input.

**Figure 5.**
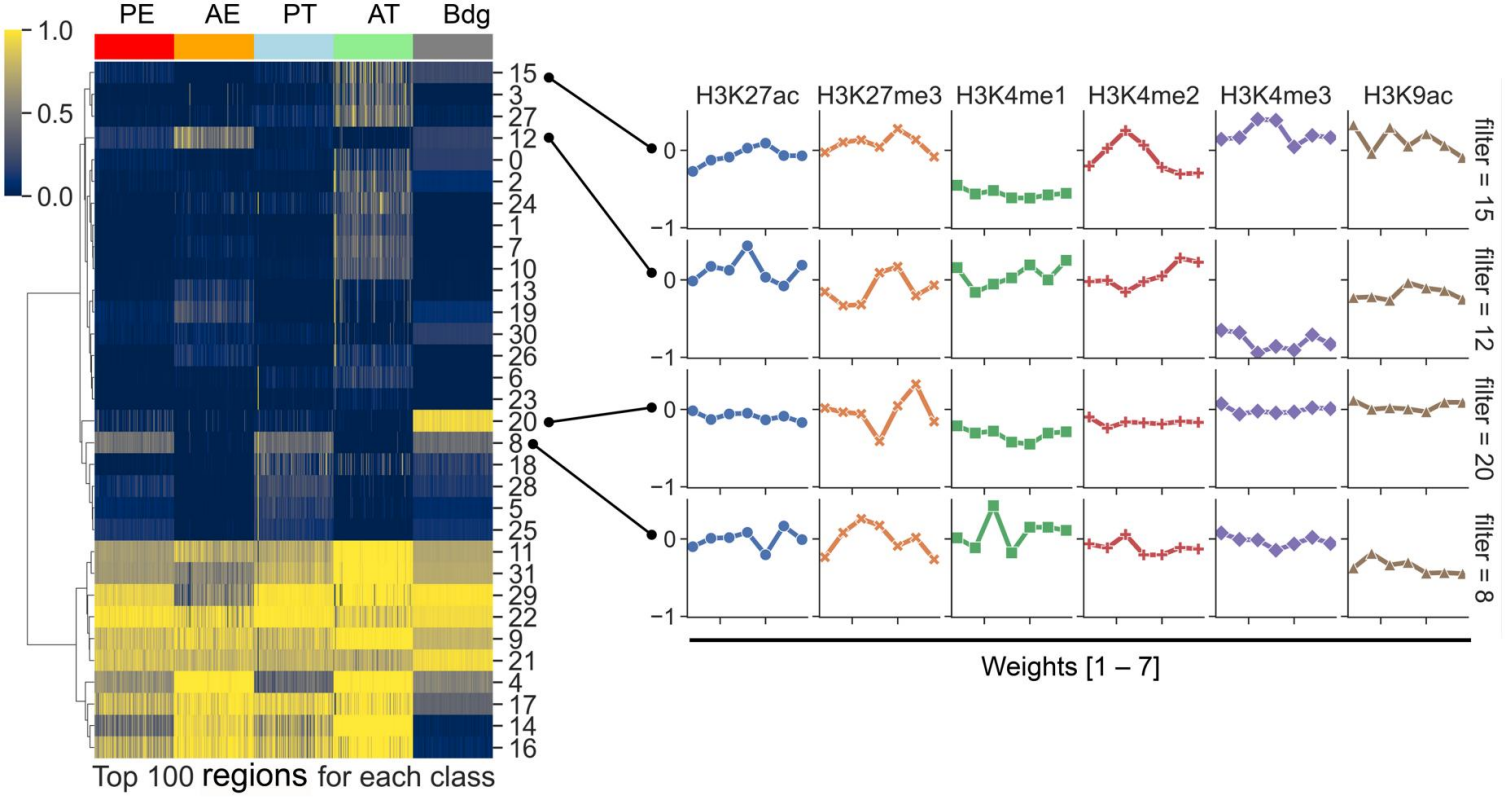
Clustering of the 32 filters of the first convolution layer of the CNN trained on K562. The top 100 regions for each of the five classes from the test set are selected by their predicted probabilities. The regions are passed through the CNN to obtain the activations for the first convolutional layer. The activations along the bin dimension is reduced to one through SVD (see main text for details) to obtain a matrix of filter × region. Each row of the matrix represents the activation pattern of a filter across the 500 regions. Hierarchical clustering is employed to cluster the rows of the matrix (left panel). Four filters are chosen which show enrichment for specific classes and the corresponding weights are depicted as line plots for six representative histone marks separately (right panel). Each row of line plots in the right panel represents the weights for the six selected histone marks of a filter, which is connected to a row in the left panel via a line with two dots on both ends.

The activation values of the first convolutional layer obtained using a single region as input is represented by a matrix of 20 × 32. Altogether, the activation values for all 500 regions were contained in a 3-D tensor of 20 × 32 × 500. Each filter’s activation values are represented by a matrix of 20 × 500. To characterize the activation patterns of the 32 filters, we applied singular value decomposition (SVD) on each 20 × 500 matrix independently to derive an eigenvector that represents the reduced activation across the 20 bins for the 500 regions, resulting in a 1 × 500 vector for each filter. By stacking the 32 eigenvectors together, this process reduced the 3-D tensor to a 2-D matrix of 32 × 500. We then employed hierarchical clustering to cluster the rows (i.e. filters) of the matrix (Fig. 5, left panel). The left panel therefore shows the reduced representation of the activation values upon passing the 500 regions through the first convolutional layer of the trained network.

The left panel of Fig. 5 shows that some filters are activated across multiple classes, which means they extract common features that are shared by the five classes. On the contrary, other filters exhibit specific activations for particular classes. To understand what has been learned in those class-specific filters, we identified four filters (15, 12, 20, 8) and depicted their weights as line plots for six representative histone marks—H3K27ac, H3K27me3, H3K4me1, H3K4me2, H3K4me3, and H3K9ac (Fig. 5, right panel). The histone marks were chosen based on our observation and the existing knowledge of chromatin biology about the association between histone marks and DREs. Filter 15 is associated with the active promoter class and contains peak detectors for H3K4me2/3, H3K27ac, and H3K9ac. Filter 12 is associated with the active enhancer class and contains a peak detector for H3K27ac and H3K4me1/2 and is deactivated with H3K4me3. Filter 20 is associated with the background class and deactivated for all histone marks except H3K27me3. Filter 8 is associated with the poised promoter, poised enhancer and background classes and contains peak detectors for H3K4me1 and H3K27me3. The feature detectors learned by the class-specific filters are consistent with the existing knowledge of the histone marks in those DREs (ENCODE Project Consortium 2012). It shall be noted that the class predictions are not driven by a single filter in the first convolution layer but rather all filters and their combinations in the following layers.

## 4. Discussion

In this study, we introduce DeepRegFinder, a pipeline for enhancer and promoter prediction in the genome using histone mark enrichment profiles. Our pipeline is powered by deep learning models and highly customizable, allowing for automated processing of ChIP-seq data. In addition to identifying promoters and enhancers, DeepRegFinder offers the ability to classify them into active and poised states, a feature not found in most other enhancer prediction tools. We conducted a comparison with existing approaches on three cell lines for enhancer and promoter predictions. DeepRegFinder consistently produced higher precision and recall scores than the other methods for both enhancers and promoters across all cell lines. To our surprise, ChromHMM produced the poorest performance among all methods, despite being one of the most popular tools to automatically annotate the genome into “chromatin states” including enhancers and promoters. This might not be an entirely fair comparison since ChromHMM is an unsupervised method that does not rely on training labels. However, the training labels for enhancer definition require only ChIP-seq data for TFs such as p300 and DHS (or ATAC-seq), which are readily available for many cell lines from the ENCODE project and other studies. Another limitation of ChromHMM is that the learned chromatin states in the emission heatmap are often ambiguous, and it can be challenging to precisely assign them to promoter and enhancer classes.

Our study also raises the important points of evaluating machine learning models properly and avoiding the common pitfalls in a machine learning study design. Both eHMM and PREPRINT report high performance in their own papers but perform poorly in our study. A closer inspection into eHMM’s algorithm revealed that eHMM’s enhancer definition used the binding profiles of H3K4me1, H3K4me3, H3K27ac, and ATAC-seq, which were later used as model input. This raises concerns of data leakage since the target is partially used as the input. In PREPRINT, statistical models are fit on the entire dataset to generate probabilistic scores that are used for model training before the dataset is split into train and test sets. This again raises concerns of data leakage. We also notice that methods are evaluated using only AUC scores in the PREPRINT study. We did not utilize AUC scores for model evaluation as they can conceal the problem of high false discovery rate (i.e. low precision) when a dataset is highly imbalanced. Instead, we used precision, recall, and mAP to evaluate models, which can better reflect the performance of a tool on a real-world genomic dataset. Additionally, PREPRINT only considers two-class classification of enhancer versus everything else. Our confusion matrix analysis shows that it’s more common for misclassifications to happen between enhancer and promoter than between enhancer and generic background. When promoter and generic background are grouped into a single negative class, the AUC is more likely to reflect the performance of enhancer versus background since it is often the largest class in a dataset. We chose to use the three-class classification problem to compare different methods because it can better illustrate a model’s ability to distinguish enhancers from promoters.

The only two methods whose performance were somewhat close to DeepRegFinder are EP-DNN and RFECS. EP-DNN uses a more traditional deep learning approach called multi-layer perceptron (MLP), which consists of about 500K parameters. However, an MLP is not a good network architecture to learn features from chromatin modification data that obviously contain spatial dependencies among adjacent genomic positions. Our CNN and RNN models contain only 26K and 12K parameters yet achieved noticeably better performance than EP-DNN. RFECS’s performance is about in line with DeepRegFinder on the three-class classification problem but falls behind on the more challenging five-class classification problem. This shows that a carefully designed deep neural network can be a very effective tool for high-throughput experimental data in genomics.

In recent years, another important progress in identifying enhancers is to use DNA sequences (Min *et al.* 2017, Yang *et al.* 2017, Kaur *et al.* 2023). The enhancer sites contain specific DNA sequences (i.e. motifs) that allow TFs to bind. A machine learning model can be trained to recognize such motifs and their combinations to predict enhancers. The DNA sequences can serve as a rich resource of information for enhancers and complement the histone mark ChIP-seq data. How to combine the DNA sequences with the ChIP-seq data requires further research. The Hi-C (High-throughput Chromosome Conformation Capture) sequencing can reveal the three-dimensional architecture of genomes (Lieberman-Aiden *et al.* 2009), which helps us understand how chromosomes fold and how enhancers physically interact with promoters. Novel machine learning methods need to be developed to model large trunks of genomic regions, or even an entire chromosome as a whole, to more accurately identify enhancers and promoters, potentially incorporating 3D chromatin interactions.

## 5. Hardware requirement and running time

Running DeepRegFinder typically takes about 2–8 h for preprocessing; 5 min for training; and 20 min for prediction on the entire human genome. Both training and prediction require only a modest GPU configuration. For this study, we ran the pipeline on a Linux system with 12 CPU cores and 32 GB memory with an NVIDIA GTX 1080 GPU with 12GB VRAM.

## Supplementary Material

vbae007_Supplementary_Data

## Data Availability

The data underlying this article are available in Github, at https://github.com/shenlab-sinai/DeepRegFinder.
