# Supplementary material for: DeepRegFinder: deep learning-based regulatory elements finder
Source: Bioinform Adv. 2024 Jan 14;4(1):vbae007. doi: 10.1093/bioadv/vbae007 (PMC10858349; doi:10.1093/bioadv/vbae007)

**Supplemental Figures**

**Fig. S1.** Confusion matrix analysis for the test set predictions across three cell lines for 3-class classification in CNN and RNN, respectively. The X-axes denote predicted labels and Y-axes denote true labels.

**Fig. S2.** Confusion matrix analysis for the test set predictions across three cell lines for 5 class classification in CNN and RNN, respectively. The X-axes denote predicted labels and Y-axes denote true labels.

**Fig. S3.** ChromHMM state emissions. Figure shows a heatmap of the emission parameters upon running ChromHMM on all three cell lines – K562, GM12878 and HepG2, where rows correspond to states and columns correspond to histone marks. The darker shade of blue indicates a higher probability of observing the corresponding mark in that state.


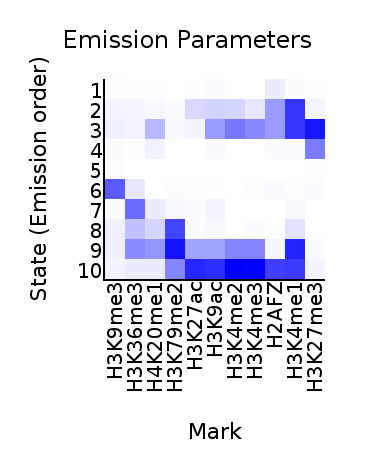

Supplement: vbae007_Supplementary_Data [file vbae007_supplementary_data.zip › SUPPL FIGURES.docx]
