# Supplementary material for: DeepRegFinder: deep learning-based regulatory elements finder
Source: Bioinform Adv. 2024 Jan 14;4(1):vbae007. doi: 10.1093/bioadv/vbae007 (PMC10858349; doi:10.1093/bioadv/vbae007)
Supplement: vbae007_Supplementary_Data [file vbae007_supplementary_data.zip › Suppl Tables.docx]

**­Tables**

**Table S1: Results of 3-class classification comparing DeepRegFinder (CNN and RNN) with five existing methods.** Precision and recall values along with mAP for enhancer and promoter are reported separately for each method.

| **Tool name** | **Cell Line** | **Region** | **Precision** | **Recall** | **mAP** |
| --- | --- | --- | --- | --- | --- |
| **DeepRegFinder (CNN)** | **K562** | Enhancer | 0.805 | 0.838 | 0.922 |
|  |  | Promoter | 0.926 | 0.871 |  |
|  | **GM12878** | Enhancer | 0.790 | 0.848 | 0.921 |
|  |  | Promoter | 0.913 | 0.889 |  |
|  | **HepG2** | Enhancer | 0.718 | 0.928 | 0.925 |
|  |  | Promoter | 0.945 | 0.886 |  |
| **DeepRegFinder (RNN)** | **K562** | Enhancer | 0.723 | 0.892 | 0.916 |
|  |  | Promoter | 0.917 | 0.860 |  |
|  | **GM12878** | Enhancer | 0.712 | 0.921 | 0.914 |
|  |  | Promoter | 0.941 | 0.859 |  |
|  | **HepG2** | Enhancer | 0.754 | 0.904 | 0.919 |
|  |  | Promoter | 0.926 | 0.886 |  |
| **EP-DNN (KimNet)** | **K562** | Enhancer | 0.672 | 0.784 | 0.842 |
|  |  | Promoter | 0.924 | 0.772 |  |
|  | **GM12878** | Enhancer | 0.697 | 0.858 | 0.882 |
|  |  | Promoter | 0.954 | 0.811 |  |
|  | **HepG2** | Enhancer | 0.762 | 0.805 | 0.889 |
|  |  | Promoter | 0.893 | 0.830 |  |
| **RFECS** | **K562** | Enhancer | 0.72 | 0.889 | 0.901 |
|  |  | Promoter | 0.926 | 0.844 |  |
|  | **GM12878** | Enhancer | 0.744 | 0.913 | 0.912 |
|  |  | Promoter | 0.94 | 0.856 |  |
|  | **HepG2** | Enhancer | 0.773 | 0.79 | 0.9 |
|  |  | Promoter | 0.908 | 0.884 |  |
| **eHMM** | **K562** | Enhancer | 0.544 | 0.255 | 0.637 |
|  |  | Promoter | 0.952 | 0.471 |  |
|  | **GM12878** | Enhancer | 0.474 | 0.381 | 0.627 |
|  |  | Promoter | 0.998 | 0.389 |  |
|  | **HepG2** | Enhancer | 0.641 | 0.278 | 0.698 |
|  |  | Promoter | 0.998 | 0.311 |  |
| **PREPRINT** | **K562** | Enhancer | 0.424 | 0.747 | 0.73 |
|  |  | Promoter | 0.992 | 0.462 |  |
|  | **GM12878** | Enhancer | 0.394 | 0.801 | 0.724 |
|  |  | Promoter | 0.997 | 0.428 |  |
|  | **HepG2** | Enhancer | 0.347 | 0.676 | 0.661 |
|  |  | Promoter | 0.994 | 0.438 |  |
| **ChromHMM** | **K562** | Enhancer | 0.364 | 0.392 | 0.493 |
|  |  | Promoter | 0.779 | 0.635 |  |
|  | **GM12878** | Enhancer | 0.376 | 0.443 | 0.505 |
|  |  | Promoter | 0.813 | 0.645 |  |
|  | **HepG2** | Enhancer | 0.208 | 0.383 | 0.43 |
|  |  | Promoter | 0.823 | 0.524 |  |

**Table S2: Results of 5-class classification comparing four models.** The precision, recall and mAP scores are presented for the four non-background classes for three cell lines. PE – poised enhancer; AE – active enhancer; PT – poised promoter; AT – active promoter.

| **Tool name** | **Cell Line** | **Region** | **Precision** | **Recall** | **mAP** |
| --- | --- | --- | --- | --- | --- |
| **DeepRegFinder (CNN)** | **K562** | PE | 0.552 | 0.624 | 0.704 |
|  |  | AE | 0.693 | 0.698 |  |
|  |  | PT | 0.652 | 0.377 |  |
|  |  | AT | 0.852 | 0.897 |  |
|  | **GM12878** | PE | 0.563 | 0.548 | 0.666 |
|  |  | AE | 0.714 | 0.807 |  |
|  |  | PT | 0.490 | 0.342 |  |
|  |  | AT | 0.915 | 0.902 |  |
|  | **HepG2** | PE | 0.702 | 0.759 | 0.707 |
|  |  | AE | 0.505 | 0.587 |  |
|  |  | PT | 0.737 | 0.680 |  |
|  |  | AT | 0.688 | 0.677 |  |
| **DeepRegFinder (RNN)** | **K562** | PE | 0.486 | 0.698 | 0.693 |
|  |  | AE | 0.663 | 0.700 |  |
|  |  | PT | 0.568 | 0.467 |  |
|  |  | AT | 0.911 | 0.834 |  |
|  | **GM12878** | PE | 0.515 | 0.597 | 0.648 |
|  |  | AE | 0.673 | 0.832 |  |
|  |  | PT | 0.450 | 0.321 |  |
|  |  | AT | 0.904 | 0.894 |  |
|  | **HepG2** | PE | 0.659 | 0.769 | 0.694 |
|  |  | AE | 0.510 | 0.409 |  |
|  |  | PT | 0.699 | 0.714 |  |
|  |  | AT | 0.718 | 0.621 |  |
| **EP-DNN**  **(KimNet)** | **K562** | PE | 0.458 | 0.325 | 0.580 |
|  |  | AE | 0.539 | 0.771 |  |
|  |  | PT | 0.625 | 0.210 |  |
|  |  | AT | 0.872 | 0.832 |  |
|  | **GM12878** | PE | 0.435 | 0.365 | 0.578 |
|  |  | AE | 0.630 | 0.827 |  |
|  |  | PT | 0.527 | 0.151 |  |
|  |  | AT | 0.903 | 0.871 |  |
|  | **HepG2** | PE | 0.648 | 0.738 | 0.657 |
|  |  | AE | 0.527 | 0.382 |  |
|  |  | PT | 0.664 | 0.627 |  |
|  |  | AT | 0.698 | 0.598 |  |
| **RFECS** | **K562** | PE | 0.525 | 0.559 | 0.68 |
|  |  | AE | 0.629 | 0.749 |  |
|  |  | PT | 0.689 | 0.347 |  |
|  |  | AT | 0.901 | 0.854 |  |
|  | **GM12878** | PE | 0.457 | 0.492 | 0.626 |
|  |  | AE | 0.643 | 0.872 |  |
|  |  | PT | 0.552 | 0.197 |  |
|  |  | AT | 0.928 | 0.868 |  |
|  | **HepG2** | PE | 0.6 | 0.796 | 0.639 |
|  |  | AE | 0.415 | 0.315 |  |
|  |  | PT | 0.668 | 0.708 |  |
|  |  | AT | 0.705 | 0.515 |  |

**Table S3: Total number of samples in each class for the 5-class classification.** List of histone marks used for each cell line are also included.

|  | **PE** | **AE** | **PT** | **AT** | **Bgd** | **No. of histone marks** |
| --- | --- | --- | --- | --- | --- | --- |
| K562 | 4313 | 6628 | 5403 | 14922 | 30000 | 12 |
|  | H2AFZ, H3K27me3, H3K4me1, H3K4me3, H3K9ac, H3K9me3, H3K27ac, H3K36me3, H3K4me2, H3K79me2, H3K9me1, H4K20me1 | | | | | |
| GM12878 | 3003 | 5529 | 3602 | 14414 | 30000 | 11 |
|  | H2AFZ, H3K27me3, H3K4me1, H3K4me3, H3K9ac, H4K20me1, H3K27ac, H3K36me3, H3K4me2, H3K79me2, H3K9me3 | | | | | |
| HepG2 | 3918 | 1224 | 12884 | 7362 | 30000 | 10 |
|  | H2AFZ, H3K27ac, H3K27me3, H3K36me3, H3K4me1, H3K4me2, H3K79me2, H3K9ac, H3K9me3, H4K20me1 | | | | | |

**Table S4: List of all BAM files used –** This table presents the ENCODE project or GEO accession IDs of BAM files used in this study.

| **Cell Line** | **Histone Mark** | **Accession ID** |
| --- | --- | --- |
| HepG2 | H2AFZ | ENCFF108XXY |
|  | H3K27ac | ENCFF805KGN, ENCFF686HFQ |
|  | H3K27me3 | ENCFF785SBQ, ENCFF247LXA, ENCFF394DQA, ENCFF030HAG |
|  | H3K36me3 | ENCFF508EQJ, ENCFF830FLY, ENCFF130PYS, ENCFF660VAM |
|  | H3K4me1 | ENCFF256HMH, ENCFF372VZP |
|  | H3K4me2 | ENCFF018EID, ENCFF837GSJ |
|  | H3K79me2 | ENCFF903AHL |
|  | H3K9ac | ENCFF583ZEK, ENCFF798OBX |
|  | H3K9me3 | ENCFF693WQC, ENCFF562AOI |
|  | H4K20me1 | ENCFF310GTD, ENCFF221MCL |
|  | GRO-seq | SRR7868785 |
| GM12878 | H2AFZ | ENCFF848PUT, ENCFF762TRA |
|  | H3K27me3 | ENCFF231DJN, ENCFF175YYN, ENCFF796DDM, ENCFF927XRX |
|  | H3K4me1 | ENCFF815TLX, ENCFF153KPG |
|  | H3K4me3 | ENCFF278QPY, ENCFF019VEK, ENCFF147ZCK, ENCFF822SQU, ENCFF396LGW, ENCFF634CBL |
|  | H3K9ac | ENCFF737GSB, ENCFF424IMO |
|  | H4K20me1 | ENCFF880XJW, ENCFF937PBY |
|  | H3K27ac | ENCFF804NCH, ENCFF948GTC |
|  | H3K36me3 | ENCFF958QVX, ENCFF460TXJ, ENCFF906JEC, ENCFF651KLD |
|  | H3K4me2 | ENCFF803ROB, ENCFF128WUO |
|  | H3K79me2 | ENCFF231YZJ, ENCFF676NDU |
|  | H3K9me3 | ENCFF663EWP, ENCFF758GUH, ENCFF370XAS |
|  | GRO-seq | SRR1552485 |
| K562 | H2AFZ | ENCFF407AUC, ENCFF472XNE |
|  | H3K27me3 | ENCFF692KQZ, ENCFF190OWE, ENCFF915XIL, ENCFF330YFF |
|  | H3K4me1 | ENCFF580LGK, ENCFF778EZR, ENCFF290LQY, ENCFF063EDR |
|  | H3K4me3 | ENCFF633WWH, ENCFF777LZD, ENCFF185YRK, ENCFF955AMI, ENCFF706SCF, ENCFF611YPB, ENCFF661UGK, ENCFF236SNL |
|  | H3K9ac | ENCFF103YPC, ENCFF698ROL, ENCFF763ZGN, ENCFF236CJR |
|  | H3K9me3 | ENCFF146NLP, ENCFF559DHZ |
|  | H3K27ac | ENCFF301TVL, ENCFF879BWC |
|  | H3K36me3 | ENCFF639PLN, ENCFF673KBG, ENCFF975JFV, ENCFF989ORU |
|  | H3K4me2 | ENCFF773VGC, ENCFF010SKB |
|  | H3K79me2 | ENCFF947DVY, ENCFF408YHI |
|  | H3K9me1 | ENCFF108HZT |
|  | H4K20me1 | ENCFF744AZG, ENCFF080MET |
|  | GRO-seq | SRR1552484 |

**Table S5: List of all BED files used –** This table presents the ENCODE project accession IDs of BED files for transcription factors or histone marks used in this study.

| **Cell Line** | **Peak Type** | **Accession ID** |
| --- | --- | --- |
| **HepG2** | H3K4me3 | ENCFF982DUT |
|  | p300 | ENCFF393LUP |
|  | DHS | ENCFF748QCZ |
| **K562** | H3K4me3 | ENCFF616DLO |
|  | DHS | ENCFF433TIR |
|  | p300 | ENCFF755HCK |
|  | CREBBP | ENCFF678FRK |
| **GM12878** | H3K4me3 | ENCFF636FWF |
|  | DHS | ENCFF073ORT |
|  | p300 | ENCFF865UDD |

**Table S6: Comparison of ChromHMM annotations with test set labels of DeepRegFinder for 3-class classification.**

| **Cell Line** | **Region** | **Precision** | **Recall** |
| --- | --- | --- | --- |
| **K562** | Enhancer | 0.439 | 0.948 |
|  | Promoter | 0.862 | 0.819 |
| **GM12878** | Enhancer | 0.413 | 0.895 |
|  | Promoter | 0.851 | 0.840 |
| **HepG2** | Enhancer | 0.310 | 0.978 |
|  | Promoter | 0.926 | 0.855 |

**Table S7: Comparison of ChromHMM annotations with test set labels of DeepRegFinder for 5-class classification.**

| **Cell Line** | **Region** | **Precision** | **Recall** |
| --- | --- | --- | --- |
| **K562** | PE | 0.212 | 0.032 |
|  | AE | 0.299 | 0.966 |
|  | PT | 0.778 | 0.019 |
|  | AT | 0.744 | 0.906 |
| **GM12878** | PE | 0.073 | 0.032 |
|  | AE | 0.317 | 0.940 |
|  | PT | 0.574 | 0.046 |
|  | AT | 0.784 | 0.915 |
| **HepG2** | PE | 0.121 | 0.087 |
|  | AE | 0.543 | 0.980 |
|  | PT | 0.539 | 0.397 |
|  | AT | 0.390 | 0.836 |

**Table S8: Validation rates of predicted promoters and enhancers using 3-class classification mode.**

| **Tool Name** | **Cell Line** | Class | Validation Rate |
| --- | --- | --- | --- |
| **DeepRegFinder (CNN)** | **K562** | Enhancer | 0.993 |
|  |  | Promoter | 0.981 |
|  | **GM12878** | Enhancer | 0.981 |
|  |  | Promoter | 0.976 |
|  | **HepG2** | Enhancer | 0.988 |
|  |  | Promoter | 0.973 |
| **DeepRegFinder (RNN)** | **K562** | Enhancer | 0.957 |
|  |  | Promoter | 0.965 |
|  | **GM12878** | Enhancer | 0.970 |
|  |  | Promoter | 0.976 |
|  | **HepG2** | Enhancer | 0.988 |
|  |  | Promoter | 0.965 |

**Table S9: Validation rates of predicted active and poised promoters and enhancers using 5-class classification mode.**

| **Tool Name** | **Cell Line** | Class | Validation Rate |
| --- | --- | --- | --- |
| **DeepRegFinder (CNN)** | **K562** | Poised Enhancer | 0.997 |
|  |  | Active Enhancer | 0.978 |
|  |  | Poised Promoter | 0.994 |
|  |  | Active Promoter | 0.993 |
|  | **GM12878** | Poised Enhancer | 0.945 |
|  |  | Active Enhancer | 0.993 |
|  |  | Poised Promoter | 0.938 |
|  |  | Active Promoter | 0.986 |
|  | **HepG2** | Poised Enhancer | 0.991 |
|  |  | Active Enhancer | 1.000 |
|  |  | Poised Promoter | 0.986 |
|  |  | Active Promoter | 1.000 |
| **DeepRegFinder (RNN)** | **K562** | Poised Enhancer | 0.957 |
|  |  | Active Enhancer | 0.978 |
|  |  | Poised Promoter | 0.941 |
|  |  | Active Promoter | 0.995 |
|  | **GM12878** | Poised Enhancer | 0.930 |
|  |  | Active Enhancer | 0.993 |
|  |  | Poised Promoter | 0.943 |
|  |  | Active Promoter | 0.965 |
|  | **HepG2** | Poised Enhancer | 0.955 |
|  |  | Active Enhancer | 0.997 |
|  |  | Poised Promoter | 0.954 |
|  |  | Active Promoter | 1.000 |

**Table S10: Comparison of DeepRegFinder test set labels with SCREEN as the baseline model.**

| Cell Line | Class | Precision | Recall |
| --- | --- | --- | --- |
| K562 | Enhancer | 0.733 | 0.619 |
|  | Promoter | 1.000 | 0.671 |
| GM12878 | Enhancer | 0.690 | 0.644 |
|  | Promoter | 1.000 | 0.691 |
| HepG2 | Enhancer | 0.703 | 0.610 |
|  | Promoter | 1.000 | 0.647 |

**Table S11: Comparison of DeepRegFinder test set labels with EnhancerAtlas as the baseline model.**

| Cell Line | Class | Precision | Recall |
| --- | --- | --- | --- |
| K562 | Enhancer | 0.354 | 0.880 |
| GM12878 | Enhancer | 0.301 | 0.901 |
| HepG2 | Enhancer | 0.201 | 0.940 |

**Table S12: Comparing 664 experimentally validated enhancer and negative control regions from Gasperini et al., 2019 with predicted enhancers from all tools utilized in this study.**


| **Tool name** | **Precision** | **Recall** | **F1** | **Total no. of enhancers recalled** | **% of non-recalled enhancer classified as promoter** |
| --- | --- | --- | --- | --- | --- |
| **CNN** | 0.985 | 0.849 | 0.914 | 564 | 92 |
| **EP-DNN** | 0.841 | 0.971 | 0.904 | 645 | 79 |
| **RNN** | 0.930 | 0.947 | 0.938 | 629 | 80 |
| **PREPRINT** | 0.909 | 0.950 | 0.929 | 631 | 61 |
| **eHMM** | 0.996 | 0.592 | 0.768 | 393 | 87 |
| **ChromHMM** | 0.915 | 0.395 | 0.601 | 262 | 96 |
| **RFECS** | 0.980 | 0.625 | 0.783 | 415 | 94 |

**Table S13: Total number of sites in EnhancerAtlas 2.0 and SCREEN database used for the analysis.**

|  | **SCREEN** | **EnhancerAtlas** |
| --- | --- | --- |
| **K562 Enhancer** | 33965 | 43168 |
| **K562 TSS** | 19320 | NA |
| **GM12878 Enhancer** | 33569 | 49615 |
| **GM12878 TSS** | 18748 | NA |
| **HepG2 Enhancer** | 28382 | 50165 |
| **HepG2 TSS** | 15974 | NA |
