# Supplementary material for: DeepRegFinder: deep learning-based regulatory elements finder
Source: Bioinform Adv. 2024 Jan 14;4(1):vbae007. doi: 10.1093/bioadv/vbae007 (PMC10858349; doi:10.1093/bioadv/vbae007)
Supplement: vbae007_Supplementary_Data [file vbae007_supplementary_data.zip › Supplemental Information.docx]

**Supplemental Information**

**DeepRegFinder: Deep Learning based Regulatory Elements Finder**

**Aarthi Ramakrishnan^1^, George Wangensteen^2^, Sarah Kim^3^, Eric J. Nestler^1^ and Li Shen^1,*^**

^1^Friedman Brain Institute and Nash Family Department of Neuroscience, Icahn School of Medicine at Mount Sinai, New York, NY 10029, USA

^2^Department of Computer Science, Brown University, Providence, RI 02912, USA

^3^Broad Institute, Cambridge, MA 02142, USA

^*^Correspondence: [li.shen@mssm.edu](mailto:li.shen@mssm.edu)

**Methods**

**Preprocessing Module:**

*Processing Promoters*

Promoters are defined using the transcriptional start sites (TSS) provided by the user in BED file format. If the mid-points of neighboring TSS sites are less than 500bp apart, the sites are merged and designated as a single TSS site. In addition, the sites are slopped to 2kb. To further define TSS specific to the cell line of interest, the sites are overlapped with peaks derived from DNA accessibility assays such as DNase I hypersensitive sites (DHS) or ATAC-seq data specific for the specific cell line. The total number of promoters are listed in Table S3.

*Processing Enhancers*

Enhancers are defined using the peak lists of enhancer-associated transcription factors (TFs), DHS and H3K4me3 specified by the user. The peak lists in the form of narrowPeak files may be obtained from ENCODE or the user shall use tools such as MACS2 to identify peaks. In this study, different TFs were used for different cell line depending on the availability – K562: CREBBP and p300; HepG2: EP300; GM12878: p300. DHS peaks are slopped to 2kb and only the TF binding sites that overlap with the DHSs are retained. Next, peaks of H3K4me3 histone mark are slopped to 2kb and *subtracted* from the retained sites. The TSS are also slopped to 2kb and subtracted from the remaining sites. The final set of sites are sorted, slopped to 2kb from their midpoints and stored as the final enhancer set. All TF BED files were obtained from ENCODE (Consortium, 2012) and the accession IDs for all samples are presented in Table S5. The total number of enhancers are listed in Table S3.

*Histone Mark ChIP-seq data collection*

Histone mark data for marks such as H3K9ac, H3K27me3, H3K4me1, H3K4me3, H3K27ac and many more can be obtained from ENCODE. Each histone mark may also have a single or multiple replicates. A script to automatically download ENCODE datasets have been included in the DeepRegFinder GitHub repository for the convenience of the user. Users can also use their own raw sequence files and do alignment to generate BAM files.

*Genomic binning and ChIP-seq processing*

Each chromosome of the genome is divided into windows of 2kb in size. Each window is further divided into 20 bins of 100bp each. Both window and bin sizes can be customized as program arguments. For each histone mark, featureCounts (Liao, et al., 2014) is utilized to compute the read coverage of each biological replicate to compute the read coverage of each genomic bin. The ENCODE accession IDs of BAM files used for this study are presented in Table S4. The read counts are normalized to Reads Per Million mapped reads (RPMs) for all bins for each replicate. Finally, the average RPMs across all replicates are computed for each bin. To define background genomic regions, 30,000 windows are randomly selected from the 2kb binned genome after excluding enhancers, TF peaks, DHS and promoter sites.

*Clustering of active and poised states using GRO-seq*

One of the main features of DeepRegfinder is that along with 2-class classification (Enhancer vs. Background) and 3-class classification (Enhancer vs. Promoter vs. Background), the pipeline also supports 5-way classification (PE, AE, PT, AT and Bgd) classification. Specifically for 5-way classification, each enhancer or promoter is classified into active or poised states based on a 2-way K-means clustering on GRO-seq data (Core, et al., 2008) derived coverage. The clustering is done using the cluster.KMeans clustering function of the sklearn package. GRO-seq data is utilized to define active or poised enhancers or promoters in the training data for 5-class classification option of DeepRegFinder. Raw GRO-seq data are processed using standard ChIP-seq pipeline to obtain BAM files, which are further processed using *featureCounts* (Liao, et al., 2014) to obtain log-normalized read counts for the defined enhancers and promoters in the genome. K-means clustering is utilized to cluster log-transformed GRO-seq read counts for enhancers into two clusters. The cluster with the larger centroid read count is designated as active while the cluster with smaller count is designated as poised. In the case of strand-specific GRO-seq data, clustering is applied separately on the sense and antisense-strand BAM files. An enhancer is classified as active or poised only when both sense and antisense labels agree on the clustering label. About 10-12% of the total enhancers were discarded due to the disagreement between sense and antisense labels in this study. The same procedure is repeated for promoters to classify them into active and poised states. In addition to GRO-seq, any kind of sequencing techniques that measure the transcriptional activity of a DRE can be used, such as PRO-seq and NET-seq.

*Defining Positive Markers*

Positive markers (PMs) serve as supporting evidence for DeepRegFinder’s whole-genome predictions to be genuine regulatory elements. If a predicted enhancer/promoter does not overlap any PM, then it is likely to be a false positive. We use PMs to estimate the validation rate of the predicted enhancers and promoters. To define PMs, we first combine multiple TFs’ binding sites with DHSs. We then choose the midpoints of these sites and slop to 2.5kb.

**Comparison of DeepRegFinder with existing tools:**

We employed DeepRegFinder’s 3-class classification mode to compare its performance with existing tools. We trained all models on chromosome 1-7 and used chromosome 8-16 as a validation set to identify hyperparameters. We then evaluated the models’ performance on chromosome 17-22, X and Y. To assess the performance of the existing tools, we compared the labels of DeepRegFinder’s test set with the predictions of the five different tools - eHMM, PREPRINT, ChromHMM, EP-DNN and RFECS on the same test set. The following outlines the data preprocessing methods used for each tool.

**eHMM**

eHMM utilizes chromatin accessibility and histone marks ChIP-seq data to predict active enhancers. The model learns the molecular structures of both promoters and enhancers, a feature absents in other tools. To compare eHMM’s performance with that of DeepRegFinder, we executed eHMM on the three cell lines. The sites in the BED files utilized to define the promoter, enhancer, and background regions for eHMM were 2kb in length, where the regions were defined in the exact same manner as in DeepRegFinder. After constructing the BED files, the *learnModel* function was applied separately on the enhancer, promoter, and background sites to generate three models, one for each class. eHMM requires BAM files for the histone marks H3K27ac, H3K4me1 and H3K4me3, along with a chromatin accessibility assay, such as DNase-seq or ATAC-seq data. We employed ATAC-seq datasets procured from ENCODE for all cell lines. The number of states for the *learnModel* function was set to 5 for enhancers and promoter and 10 for background. Next, we executed the *constructModel* function to create a consolidated model utilizing the three models obtained for each class. Lastly, the *applyModel* module utilized the unified model to generate predictions on the BED file that encompasses 2kb regions from chromosome 17-22, X and Y.

To compare the predicted enhancer sites by eHMM with the test set enhancer sites of DeepRegFinder, we utilized the “bedtools intersect” command to calculate precision and recall scores. We computed precision by determining the number of sites that overlap between the eHMM enhancer predictions and the DeepRegFinder enhancer test set sites, and divided that by the total number of eHMM predictions that intersect with DeepRegFinder's test set. Recall was determined by calculating the number of sites that overlap between the eHMM enhancer predictions and DeepRegFinder enhancer test set sites, and dividing that by the total number of enhancer sites in the DeepRegFinder test set. We repeated the aforementioned process for the promoter class.

**PREPRINT**

PREPRINT predicts genome-wide enhancer sites using a Support Vector Machine classifier with a Gaussian kernel. Before PREPRINT could be executed on our dataset, it was necessary to make several modifications to the script. For instance, modifications need to be made to ensure the training dataset only includes chromosome 1-7 and the test dataset only includes chromosome 17-22, X and Y. Further, the pipeline was modified to use the hg38 as opposed to the hg19 reference genome. Certain steps that deal with automatically downloading the raw FASTQ files originally used in the study from ENCODE and aligning them to the reference genome were removed, as we used existing BAM files as input. PREPRINT is originally designed to conduct a 2-class classification on the test set to distinguish enhancers from non-enhancers. In order to adapt to our needs of 3-class classification, we made modifications on the model to produce 3-class outputs.

We retrieved the DNase and P300 peak lists for K562, GM12878, and HepG2 from the ENCODE database. Promoter site annotations were obtained from GENCODE for human genome version hg38. To run the workflow on each cell line, the file config.yaml file was edited to include path to the folder containing BAM files of all histone marks. The file samples.tsv was edited to include the names of all BAM files. The workflow was initialized using the command “snakemake” following the activation of the Anaconda environment. Training and test datasets were generated using the script 2_make_profiles.R. The script 3_train_predict.R was modified to accept two .RDS files – one for train and the other for test. A model was trained on the training data and predictions were made using the trained model on the test data. The predicted labels were saved to an external CSV file, which were further used for evaluation with the test set labels of DeepRegFinder.

**ChromHMM**

ChromHMM is a tool based on multivariate Hidden Markov Model used for characterizing chromatin states in the genome. This tool uses ChIP-seq datasets of histone modifications to find distinct patterns or states in the genome, which the user further manually annotates as enhancers, promoters, or other regions.

The three modules in ChromHMM used for the analysis were *BinarizeBam*, *LearnModel* and *MakeSegmentation*. Upon running *BinarizeBam* on BAM files from cell lines K562, GM12878 and HepG2, we obtained multiple text files: one for each chromosome and cell line which consisted of binary data indicating the presence or absence of reads for histone marks of interest in each of the 200bp bins of the genome. The bin length can be changed by the user. We generated train and test datasets using the text files for chromosome 1-7 for training and chromosome 17-22, X and Y for test set. Next, we ran the function *LearnModel* which reads all files associated with the training data and outputs a model trained on this data. We used 10 states for training as recommended in their manual (<https://ernstlab.biolchem.ucla.edu/ChromHMM/>) and we obtained a text file containing the model parameters. Finally, we ran the module *MakeSegmentation*. This module requires the user to specify the model filename as well as the name of the folder containing the test set. The output of this module was one BED file for each cell line containing coordinates covering the entire chromosome and their assigned states ranging from 1-10 (See Fig. S3). State E2 was annotated as enhancer as this state is enriched for the histone mark H3K4me1 and depleted for H3K4me3. State E10 was annotated as promoter as this state is enriched for the histone marks H3K4me3 and H3K9ac. The evaluation of results was carried out by computing precision and recall values similar to eHMM and PREPRINT.

Since ChromHMM is an unsupervised method, we hypothesize that it may need a large amount of training data to obtain decent performance. To get a more comprehensive understanding of ChromHMM’s performance, we downloaded publicly available chromatin states generated by ChromHMM trained on 127 cell lines from the UCSC Genome Browser for the three cell lines of interest. Annotations for the human genome were available as BED files at https://egg2.wustl.edu/roadmap/web_portal/chr_state_learning.html. For each site in the test set, the corresponding annotation was obtained from the BED file using “bedtools closest” (Quinlan and Hall, 2010). Finally, the annotations from both sources were compared and precision and recall scores were calculated for the overlap. Table S6 and S7 list precision and recall values for 3-class and 5-class classifications across the three cell lines.

**RFECS**

RFECS is based on the Random Forest algorithm. We implemented the RFECS algorithm in Python using the *RandomForestClassifier* module from the scikit-learn package (<https://scikit-learn.org/>). We converted the PyTorch tensors for the training, validation and test sets into *NumPy* arrays to use with the *RandomForestClassifier* module. The number of estimators was set to be 65. *max_features* was set to “sqrt” and *class_weight* was set to “balanced”. All these hyperparameters are the same as in the original paper.

**EP-DNN**

EP-DNN is a multilayer perceptron implemented in PyTorch and integrated into the DeepRegFinder pipeline. To use EP-DNN, users can set the *net_choice* parameter in training_data.yaml file to “kimnet”. The model architecture consists of three linear layers with 600, 500 and 400 neurons, respectively. Each layer is followed by a softplus activation function. Additionally, the network contains a dropout layer with a dropout rate of 0.5. The final layer is a linear layer with output neurons set to “nb_cls” and a softmax activation function is applied. EP-DNN uses SGD as the optimizer with *momentum* set to 0. The weight decay was set to 0.0001and the initial learning rate was set to 0.01.

Pre-processing of datasets for running EP-DNN is carried out using the pre-processing module of DeepRegFinder. Precision and recall values were computed using the *precision_recall_fscore_support* method of the scikit-learn package (https://scikit-learn.org /) for the test set labels against the predicted probabilities.

References

Consortium, E. P. (2012, Sep 6). An integrated encyclopedia of DNA elements in the human genome. *Nature, 489*(7414), 57-74. <https://doi.org/10.1038/nature11247>

Dahl, G. E., Yu, D., Deng, L., & Acero, A. (2012). Context-Dependent Pre-Trained Deep Neural Networks for Large-Vocabulary Speech Recognition. *IEEE Transactions on Audio, Speech, and Language Processing, 20*, 30-42.

Hochreiter, S., & Schmidhuber, J. (1997, Nov 15). Long short-term memory. *Neural Comput, 9*(8), 1735-1780. <https://doi.org/10.1162/neco.1997.9.8.1735>

Ioffe, S., & Szegedy, C. (2015). *Batch Normalization: Accelerating Deep Network Training by Reducing Internal Covariate Shift* Proceedings of the 32nd International Conference on Machine Learning, Proceedings of Machine Learning Research. <http://proceedings.mlr.press/v37/ioffe15.html>

Kim, S. G., Harwani, M., Grama, A., & Chaterji, S. (2016, Dec 8). EP-DNN: A Deep Neural Network-Based Global Enhancer Prediction Algorithm. *Sci Rep, 6*, 38433. <https://doi.org/10.1038/srep38433>

Kingma, D. P., & Ba, J. (2014). Adam: A method for stochastic optimization. *arXiv preprint arXiv:1412.6980*.

Liao, Y., Smyth, G. K., & Shi, W. (2014, Apr 1). featureCounts: an efficient general purpose program for assigning sequence reads to genomic features. *Bioinformatics, 30*(7), 923-930. <https://doi.org/10.1093/bioinformatics/btt656>

Nair, V., & Hinton, G. E. (2010). Rectified linear units improve restricted boltzmann machines. Icml,
